# The Design and validation of a Nursing Plan for Elderly Patients with Postoperative Delirium

**DOI:** 10.3390/ijerph16224504

**Published:** 2019-11-15

**Authors:** Estela Melguizo-Herrera, Ana Acosta-López, Isabel Patricia Gómez-Palencia, Yolima Manrique-Anaya, César Hueso-Montoro

**Affiliations:** 1 Faculty of Nursing, University of Cartagena, Cartagena de Indias 130001, Colombia; emelguizoh@unicartagena.edu.co (E.M.-H.); aacostal@unicartagena.edu.co (A.A.-L.); ipgomezp@unicartagena.edu.co (I.P.G.-P.); ymanriquea@unicartagena.edu.co (Y.M.-A.); 2 Faculty of Health Sciences, University of Granada, 10816 Granada, Spain

**Keywords:** delirium, postoperative period, nursing process

## Abstract

Delirium is the sudden alteration of the state of consciousness and perception, fluctuating over hours or days. It predominates in older adults and is associated with the aging process. The incidence of delirium increases between 10% and 15% in surgical interventions. The objective of this study was the design and validation of a nursing care plan for elderly patients with postoperative delirium. This study was based on the Delphi method and applied to nursing professionals at the Hospital Universitario del Caribe, Cartagena. The sample consisted of 36 nurses with knowledge of the taxonomy of nursing diagnoses. The care plan was applied in two rounds. For the analysis, measures of central tendency and dispersion were used, as well as frequency and percentages. The participants were women (90.9%) from the hospitalization service (51.5%), with training in Nursing Diagnosis (NANDA), Nursing interventions classification (NIC) and Nursing Outcome Classifications (NOC) (78.8%). The validated care plan has eight diagnostic features. Highlights include “Risk for Ineffective Cerebral Tissue Perfusion” and “Disturbed Sleep Pattern” (in 98.1%; 11 results), with the highest score in the first round being “Vital Signs” (with 100%) and “Sleep” (100%) and “Mobility” (100%) in the second round. Forty-four interventions and 18 suggested activities were identified. This care plan offers the nursing professionals reliable and pertinent tools in clinical practice for the management of patients with postoperative delirium.

## 1. Introduction

Delirium is known to cause sudden alterations in attention and state of consciousness. Delirium’s severity tends to fluctuate over hours or days [1] and can occur at any age, but it is more frequent in older adults due to its association with the aging process, recurrent medical illnesses, polypharmacy, and surgical procedures [2,3]. An age over 65 has been described as a risk factor for postoperative delirium [4].

Studies show that the incidence of delirium increases between 10% and 15% in surgically treated patients and for every 48 h with delirium, mortality increases by 11% [5]. Previous studies show that an increase in delirium partly occurs because it goes unnoticed by more than 67% of non-psychiatric doctors, and only 22% of nursing staff can define delirium and its diagnostic criteria [6,7]. In addition, the detection and treatment of delirium create an additional workload for the nursing team [8].

In the operating room, medical–surgical hospitalization, and Intensive Care Units of the Hospital Universitario del Caribe (HUC) (Cartagena, Colombia), in charge of elderly patients, we identified a lack of diagnosis and timely management of delirium. From direct interviews with representatives of different disciplines, it was evident that practitioners do not know the multidimensional impacts of delirium, how to identify it, assess it, prevent it, manage it, or reduce its intensity once it is present, as recommended by the scientific literature [9]. This result is contrary to what would be expected based on the training in the management of nursing diagnoses that is required to work in a hospital, as well as the work dynamics in each area observed by researchers at the time of their care practice.

Consequently, it became evident that we must elaborate a nursing care plan that allows the early diagnosis of delirium through nursing assessment in the first 72 h after surgery, as well as the application of the nursing care process—a tool that allows nurses to provide and plan care based on the applicability of Evidence-Based Nursing adapted to the nursing taxonomy, Nursing Diagnosis (NANDA), Nursing interventions classification (NIC) and Nursing Outcome Classifications (NOC) [10,11].

The standardization of diagnoses, objectives, and interventions established by the NANDA International (NANDA-I) includes health problems and vital or potential processes treated by nursing based on clinical judgment. NOC are global and standardized classifications used to evaluate the results of nursing interventions; NIC is understood as the center of interest and a global and standardized classification for nursing interventions, which are classified as priority, probable, or suggested interventions [12,13].

In the pursuit of ideas to achieve standardized care of delirium, the scientific literature shows studies that have applied NANDA-NOC-NIC, which has facilitated advancement in the language of nursing professionals [14,15].

Based on the above, the objective of this study was to design a nursing care plan based on the NANDA-NOC-NIC taxonomy for older adults with postoperative delirium.

## 2. Materials and Methods 

As in previous research, a systematic review was carried out to identify nursing interventions for older adults with postoperative delirium. These interventions were grouped into three lines of care: the assessment of cognitive status, non-pharmacological interventions, and pharmacological interventions. These three lines were taken as the basis for the development of the nursing plan. 

NANDA diagnoses that were related to the results of the review were first identified. Later, using the interrelationship between the NANDA-NIC_NOC nursing languages, the NIC and NOC were determined. Finally, other interventions that were considered relevant after the review were identified, but these interventions were not included in the NIC taxonomy. The first care plan was then designed; this plan consisted of 8 NANDA diagnoses, 14 NOC, 26 NICs, and 53 suggestions.

For the validation, the Delphi method was chosen. This method seeks to systematically collect judgments from a group of experts on a specific situation and organize, analyze, and measure the information obtained until a general agreement is reached. A 70% agreement among judges is considered the minimum score for validation of the items [16]. This approach has been very useful in the field of health sciences, enabling a greater understanding of reality from different perspectives and reaching an agreement on issues of interest based on expert opinions and valid statistical measures [17,18].

The population under consideration were the professional nursing staff of the operating area, medical–surgical hospitalization, emergency, and Intensive Care Units of the HUC. This sample consisted of 36 professionals, which complies with the criterion established in the literature [16]. As an inclusion criterion, it was determined that the professional must have had a minimum service length of one year in any of the units included in the study.

Intentional sampling was carried out at the work site of the participants. The first round of data collection was in a group; the purpose of the study was then explained the informed consent was completed. During the second round, feedback was given to the experts, giving them the scores obtained by each NANDA-NOC-NIC suggested during the first round, so they could ratify their agreement or disagreement with them. When there was a disagreement with an item, the participants justified their opinion by proposing new options within the care plan, which were later discussed by the researchers. After this second round, the final instrument was designed.

The experts evaluated the NANDA, NOC, and NIC suggestions using a Likert-type scale with the following ratings: 1 = strongly disagree, 2 = disagree, 3 = undecided, 4 = agree, and 5 = strongly agree. This process obtained objective results that were numerically integrated. A NANDA, NOC, or NIC was considered approved when at least 70% of its qualifications were between 4 or 5.

The results of each session were organized in a database in Microsoft Excel, version 2013 (Microsoft, Washington, USA). Statistical analyses were conducted using the Statistical Package for the Social Sciences (SPSS) program, version 22, (IBM, New York, USA, for Windows). Descriptive statistics were computed by calculating the mean, standard deviation, frequencies, and percentages.

In order to guarantee the quality of the results in the use of this Delphi methodology, in the formulation of the problem, we took care to choose a care plan resulting from scientific investigation. In the selection of experts, we selected professionals with management of the subject. The elaboration and application of the questionnaires, as well as the analysis of the results, was carried out by researchers in consensus.

The ethical aspects were also considered, so the voluntary participation of the subjects was considered and the informed consent was completed [19,20]. Before this study was carried out, approval was obtained from the Research Committee of the Faculty of Nursing of the University of Cartagena (act No. 08 of August 18, 2016).

## 3. Results

Thirty-six nurses participated in the first round and 33 participated in the second round. Of these nurses, 90.9% (30) were women aged between 22 and 60 years, with a mean age of 37.6 years (SD = 11.6). These nurses worked in hospitalization 51.5% (17), emergencies 30.3% (10), surgery 6.1% (2); they also worked in external consultation, coordination, surgery, and the Intensive Care Unit (3%). A total of 78.8% (26) stated that they had been trained in the application of care plans, and 18.2% (6) had specialized.

The first instrument was modified by the experts in the three care lines according to the degree of dispersion of the responses between 25, 50, and 75, which showed changes in the NOC results, NIC interventions, and in the suggested interventions, producing the second plan of care as a result. This was discussed by the investigators when observing that the activities suggested by the literature are included in taxonomy interventions. This approach required exhaustive work when comparing each of the suggested activities with the taxonomy to give a code for the intervention. This highlights that many of the suggested interventions may be developed into activities for each NIC, but we decided not to include these so as not to make the care plan more complex. It is important that the suggested interventions guide nurses in the development of activities to be carried out in each NIC. Finally, of the 38 suggested activities, 20 were included in the taxonomy. An example of this process is shown in Table 1.

With the previous exercise, the NICs increased to 44; those suggested without a code were left in the NIC box.

During the first and second sessions, the eight nursing diagnoses that were proposed in the care plan were maintained (Table 2).

The diagnoses with the highest percentage of acceptance were: “Risk for Ineffective Cerebral Tissue Perfusion” and “Disturbed Sleep Pattern”, with 98.1%. The lowest score was obtained by “Imbalanced Nutrition: Less than Body Requirements”, with 90.05%; the latter in the second round obtained the highest score, with 100% acceptance. In the second round, the diagnosis that obtained the lowest percentage of acceptance was “Decrease Cardiac Output”, with 87.7%. It was not necessary to eliminate any diagnoses.

Changes to the NOC results are presented by acceptance score for those ultimately remaining in the care plan (Table 3).

The eliminated NOCs include “Distorted Thought Self-Control”, “Cognitive Orientation”, and “Neurological Status”; those related to the suggested NOCs include facilitating the use of lenses and/or hearing devices, haloperidol at low doses, and treatment with morphine.

Ultimately, the final care plan designed with the application of the Delphi method was consolidated (Table 4).

## 4. Discussion

This research provides a nursing care plan including eight NANDAs, 11 NOCs, 44 NICs, and 18 suggested interventions. The nursing care has been validated through the application of the Delphi methodology. The literature [21,22] shows that the management of delirium in medicine focuses more on diagnosis and clinical treatment, while in nursing, activities for the care of a person predominate. Professionals perform general activities for delirium management without considering those activities for frequent diagnosis postsurgery [22]. This difficulty in management is related to its multifactorial nature or to the lack of prevention and/or treatment strategies to be implemented [23]. Therefore, the plan of care is a strategy where professionals in nursing intervene based on the specific needs of health situations (e.g., delirium).

Previous studies [24,25] have shown that specialists in critical medicine are not clear about the definition of delirium, do not use validated tools to detect delirium, and do not have adequate knowledge for its prevention and management. Similarly, another study [26] mentions that the knowledge of nursing professionals regarding the manifestation of delirium is superficial, which has a direct impact on the practices carried out for the prevention and monitoring of delirium. Consequently, having a validated care plan with a standardized language will strengthen the dynamics of care in the aforementioned deficit areas.

This study highlights the importance of training plans for nurses and family members in their approaches to delirium, showing that the educational programs offered to health professionals allow them to influence clinical decision making when identifying risk factors and diagnoses and, in turn, guide the prevention and treatment to be implemented [27]. The use of a technique like the ABCDE bundle affects the promotion of a comfortable and safe environment for the patient [28].

To strengthen the above measures, it is necessary to have protocols, guidelines, and care plans, as well as trained personnel for the management of delirium, which will lead to a shorter stay in the ICU and a decrease in episodes of postsurgical delirium [29].

The implementation of this care plan not only responds to post-surgical management but, according to the literature, allows activities that facilitate the prevention of delirium via the implementation of tools built on the basis of scientific evidence [30,31]. The above is similar to another study, where 20 experts structured the aspects to be considered in the management of delirium and contemplated these aspects based on the standards of quality and safety in health care; the proposed interventions, such as education, training, comprehensive evaluation, family aspects, individualized attention, and multidisciplinary participation [32], are all shared in the plan of care validated in this study.

It should be noted that, in nursing care plans, there are abundant non-pharmacological measures for the management of delirium. The use of non-pharmacological therapies with strategies that have multiple components have reduced incidents in patients (up to 50%) with delirium [33], resulting in reduced costs for health institutions [34].

Similarly, other authors [27,31] have confirmed that nursing is more involved with non-pharmacological measures, which is related to the findings of the present study. The management of cognition, sleep, and comfort stands out among the interventions identified, which is consistent with other research that provided preventive activities in the management of delirium [35].

Other studies also highlight the relevance of using nurse taxonomies in delirium care, showing that that such taxonomies lead to concise, prompt, and reliable care, thereby resulting in faster recovery [36] and allowing a comprehensive management approach with trained team dynamics, where interventions like education, training, early mobilization, and individualized attention are important aspects in patient care and for institutions that manage geriatric patients [32].

Therefore, this study, when obtaining a tool for the nursing process and an application of the specific taxonomy for the management of delirium, achieves nurse–patient–diagnostic interaction and identifies vital elements, such as the use of nonverbal communication focused on writing, signals, and technological devices, that allow a clinician to keep the patient alert and attentive and maintain their orientation in time and place, thereby reducing an increase in their delirium [37]. Similar to the results found by another study with the use of Q methodology, through the subjectivity of expert nurses, four aspects that occur in adult patients with delirium were shown, thereby illustrating typical diagnostic patterns, a recognition of signs and symptoms prior to diagnosis, continuous observations of the patient at risk, and an evaluation of the diagnosis with the use of instruments that add to the expertise of the nursing professional [38].

The limitations found during the study were related to the difficulty in filling the instrument by the nurses who participated, which made it necessary to have a personalized job in the second round, which lengthened the initial study period. On the other hand, this study was carried out in a single center, and the results cannot be directly extrapolated to other populations.

## 5. Conclusions

The objective of this work was to design and validate a nursing care plan based on the NANDA-NOC-NIC taxonomy in patients with postoperative delirium. As a result, a plan based on eight NANDA diagnoses, 11 NOCs, 44 NICs, and 18 suggested activities is provided. The “Risk for Ineffective Cerebral Tissue Perfusion” or “Disturbed Sleep Pattern” are relevant as central diagnoses. “Vital Signs”, “Sleep”, “Mobility” and “Respiratory Status: Airway Patency” are relevant as NOC indicators. Highlighted NICs include “Delirium Management”, “Fluid/Electrolyte Management”, “Vital Signs Monitoring”, “Nutrition Management”, “Exercise Promotion”, “Pain Management”, and “Medication Administration”.

This nursing care plan aims more at the prevention than the treatment of delirium, so we believe that this care plan has applicability not only from a post-surgical perspective, as originally proposed, but also from the perspective of prevention.

## Figures and Tables

**Table 1:** Examples of suggested interventions that were contained in the nursing interventions classification (NIC).

First Session				Second Session			
Item	X	M	%	Item	X	M	%
Assessment of the state of consciousness using the Glasgow scale.	4.6	5	100	(2620) Neurologic Monitoring	4.2	4	87.1
				(6440) Delirium Management	4.3	4	95.8
Levels of hematocrit and hemoglobin. Take a laboratory specimen and request a reservation and cross-check if necessary.	4.4	5	96.4	(7690) Laboratory Data Interpretation	4.2	4	88
Electrolyte control	4.4	5	98.8	(2080) Fluid/Electrolyte Management	4.5	5	100
Administration of blood products according to need.	4.3	4	96.8	(4030) Blood Products Administration	3.6	4	77.9
Monitoring of vital signs.	4.6	5	100	(6680) Vital Signs Monitoring	4.4	5	95.9
Supplementary nutrition.	4.4	4	98.1	(1100) Nutrition Management	4.2	4	100
Coordinate with nutrition for the protein quality and quantity required by the patient.	4.3	4	91.7	(5246) Nutrition Counseling	4.3	4	95.8
Walk; be out of bed.	4.3	4	96.1	(0200) Exercise Promotion.	4.5	5	98
Visualize the facial expression of pain.	4.6	5	96.4	(1400) Pain Management	4.5	5	98
Monitor adverse reactions to medications.	4.7	5	98.3	(2300) Medication Administration	4.5	5	100

X: mean M: median.

**Table 2:** Nursing Diagnosis (NANDA) result of the first and second session.

Items	Sessions					
	First			Second		
	X	M	%	X	M	%
(00128) Acute Confusion.	4.3	4	92.1	4.3	4	92.3
(00029) Decrease Cardiac Output.	4.2	4	94.6	4.1	4	87.7
(00201) Risk for Ineffective Cerebral Tissue Perfusion.	4.4	4	98.1	4.5	5	98
(00002) Imbalanced Nutrition: Less than Body Requirements.	4.1	4	90.5	4.5	5	100
(00198) Disturbed Sleep Pattern.	4.5	5	98.1	4.5	5	96
(00085) Impaired of Physical Mobility.	4.4	4	96.8	4.5	5	94
(00132) Acute Pain.	4.4	4	94.3	4.5	5	98
(00032) Ineffective Breathing Pattern.	4.5	5	96.3	4.5	5	96

X: mean M: median.

**Table 3:** Nursing Outcome Classifications (NOC) results result of the first and second session.

Items	Sessions					
	First			Second		
	X	M	%	X	M	%
(0900) Cognition.	4.3	4	92.9	4.2	4	93.7
(0401) Circulatory Status.	4.2	4	92.5	4.2	4	87.1
(0400) Cardio Pump Effectiveness.	4.2	4	90.4	4.1	4	86
(0601) Fluid Balance.	4.2	4	96	4.4	5	93.9
(0802) Vital Signs.	4.6	5	100	4.5	5	98
(0600) Electrolyte & Acid/Base Balance.	4.4	4.5	96.2	4.5	5	98
(1009) Nutritional Status: Nutrient Intake.	4.4	4	96.7	4.4	4	98
(0004) Sleep.	4.4	4	98.1	4.6	5	100
(0208) Mobility.	4.4	4	98.1	4.4	4	100
(1605) Pain control.	4.6	5	96.9	4.4	5	93.9
(0410) Respiratory Status: Airway Patency.	4.4	4	97.8	4.6	5	100

X: mean M: median.

**Table 4:** Nursing Care Plan for the management of delirium to elderly adults in postoperative.

Nursing Diagnosis (NANDA)	Nursing Outcome Classification (NOC)	Nursing Interventions Classification (NIC)
Domain 5. Perception/Cognition. Class 4. Cognition. (00128) Acute Confusion. Related factors: Over 60 years of age, Fluctuation in sleep-wake cycle, Delirium.	(0900) Cognition.	(6440) Delirium Management.
		(2620) Neurologic Monitoring.
		(5390) Self-Awareness Enhancement
		(5612) Teaching: Prescribed Exercise.
		(4720) Cognitive Stimulation.
		(4976) Communication Enhancement: Speech Deficit.
		(4974) Communication Enhancement:Hearing Deficit.
		Application of Confussion Assessment Method (CAM) scale confussion assessment method.
Domain 4. Activity/Rest. Class 4. Cardiovascular/Pulmonary Responses. (00029) Decrease Cardiac Output. Related factors: Altered heart rate, Altered stroke volume, Altered preload, Altered afterload, Altered contractility.	(0401) Circulation Status.	(6680) Vital Signs Monitoring.
		(3480) Lower Extremity Monitoring.
		Maintain average blood presure at 80 mmHg.
		Remove invasive devices in the minimum possible time. During the moment of the alteration of the state of consciousness, patients can tend to withdraw devises.
	(0400) Cardio Pump Effectiveness.	(4040) Cardiac Care.
		(4030) Blood Products Administration.
		(4235) Phlebotomy: Cannulated Vessel.
		(7820) Specimen Management.
		(4150) Haemodynamic Regulation.
		(7690) Laboratory Data Interpretation.
		Monitoring hematocrit and hemoglobin levels.
		Volumes transfused during surgery, no greater than 1000 cc, and/or according to the patient’s requirements.
Domain 2. Nutrition. Class 1. Ingestion. (00002) Imbalanced Nutrition: Less than Body Requirements. Related factors: Biological factors, Psychological factors, Inability to digest food, Inability to ingest food.	(1009) Nutritional Status: Nutrient Intake.	(1160) Nutritional Monitoring.
		(1120) Nutritional Therapy.
		(5246) Nutritional Counseling.
		(1100) Nutrition Management.
		(7690) Laboratory Data Interpretation.
		(1803) Self-Care Assistance: Feeding.
		(2620) Neurologic Monitoring.
		Motivate the patient to eat at the auxiliary table.
		Take spicemen for nutritional assessment.
		Allow accompanying family members during meal times.
Domain 4. Activity/Rest. Class 1. Sleep/Rest. (00198) Disturbed Sleep Pattern. Related factors: Change in daylight-darkness exposure, Noise, Lack of sleep privacy.	(0004) Sleep.	(1850) Sleep Enhancement.
		(5880) Calming Technique.
		(6480) Environmental Management.
		(6482) Environmental Management: Comfort.
		(4410) Mutual Goal Setting.
		(4400) Music Therapy
		Avoid, as far as possible, the use of sedative medication.
		Reduce sleep during the day to short naps.
		Stimulate physical and mental exercise during the day.
Domain 4. Activity/Rest. Class 2. Activity/Exercise. (00085) Impaired of Physical Mobility. Related factors: Cognitive impairment, Sensoriperceptual impairments, Neuromuscular impairment, Pain.	(0208) Mobility.	(0840) Positioning.
		(0221) Exercise Therapy: Ambulation.
		(0224) Exercise Therapy: Joint Mobility.
		(0200) Exercise Promotion.
		(4310) Activity Therapy.
		(6486) Environmental Management: Safety.
Domain 4. Activity/Rest. Class 4. Cardiovascular/Pulmonary Responses (00201) Risk for Ineffective Cerebral Tissue Perfusion.	(0601) Fluid Balance. (0802) Vital Signs. (0600) Electrolyte and Acid/Base Balance.	(2080) Fluid/Electrolyte Management.
		Look out permeability of the venous access pathway.
Domain 12. Comfort. Class 1. Physical Comfort. (00132) Acute Pain. Related factors: Injury agents (biological, chemical, physical)	(1605) Pain Control.	(2210) Analgesic Administration.
		(2300) Medication Administration.
		(2380) Medication Management.
		(1400) Pain Management.
		(1480) Massage.
		(3350) Respiratory Monitoring.
		Explain to the patient what medication is going to be administered and what it is for.
		Remove invasive catheterization in the minimum possible time.
		Facilitate accessibility of the call bell in case of need of the patient.
		Monitoring oxygen saturation and administer oxygen therapy 3–4 L/min.
Domain 4. Activity/Rest. Class 4. Cardiovascular/Pulmonary Responses. (00032) Ineffective Breathing Pattern. Related factors: Anxiety, Body position that inhibits lung expansion, Pain, Neurological damage, Respiratory muscles fatigue.	(0410) Respiratory Status: Airway Patency.	(3390) Ventilation Assistance.
		(3230) Chest Physiotherapy.
		(3320) Oxygen Therapy.
		Cleaning of the airways for necessary reason.
		Keep in fowler, if possible.
